# Variation in Leaf Functional Traits of *Populus laurifolia* Ldb and *Ulmus pumila* L. Across Five Contrasting Urban Sites in Ulaanbaatar, Mongolia

**DOI:** 10.3390/plants13192709

**Published:** 2024-09-27

**Authors:** Otgonsaikhan Byambasuren, Anujin Bat-Amgalan, Ser-Oddamba Byambadorj, Jonathan O. Hernandez, Tuguldur Nyam-Osor, Batkhuu Nyam-Osor

**Affiliations:** 1 Laboratory of Forest Genetics and Ecophysiology, National University of Mongolia, Ulaanbaatar 14201, Mongolia; saikhnaa80@gmail.com (O.B.); seroddamba@gmail.com (S.-O.B.); nttuguldur0810@gmail.com (T.N.-O.); 2 Department of Biology, School of Arts and Sciences, National University of Mongolia, Ulaanbaatar 14201, Mongolia; anubat0424@gmail.com; 3 Department of Forest Biological Sciences, College of Forestry and Natural Resources, University of the Philippines, Los Baños 4031, Philippines; johernandez2@up.edu.ph; 4 Institute of Geography and Geoecology, Mongolian Academy of Sciences, Ulaanbaatar 15170, Mongolia

**Keywords:** chlorophyll concentration, leaf performance index, phenotypic plasticity index, plant traits, urban trees

## Abstract

Amid urbanization, studying leaf functional traits of woody plants in urban environments is essential for understanding how urban green spaces function and how they can be effectively managed sustainably. In this study, we investigated the effects of different growing conditions on the morpho-physiological traits of *Populus laurifolia* and *Ulmus pumila* across five contrasting urban sites. The leaf area (LA), leaf length (LL), leaf width (LW), leaf biomass (LB), specific leaf area (SLA), leaf chlorophyll concentration, chlorophyll fluorescence parameters, leaf water potential at predawn (Ψ_pd_) and midday (Ψ_md_), leaf performance index (PI_abs_), and phenotypic plasticity index (PPI) were compared across five contrasting urban sites. The soil chemical and physical properties were also compared between sites. There were significant differences in soil physicochemical characteristics between sites. We found significant effects of site on most of the morpho-physiological traits measured, except for Ψ_md_. The leaf chlorophyll concentration of *P. laurifolia* and *U. pumila* varied significantly between sites. The Ψ_pd_ was significantly different between years and sites. In *U. pumila*, the mean PPI for morphological traits (0.20) was lower than that for physiological traits (0.21). In conclusion, we revealed significant variations in the morpho-physiological traits of *P. laurifolia* and *U. pumila* across the five urban sites. Hence, long-term, large-scale studies are recommended to examine how diverse species respond to different urban growing conditions and to include other ecologically important plant traits for a better understanding of urban trees in a changing environment.

## 1. Introduction

The trend of urbanization is rapidly increasing, with more than half of the world’s population today now living in urban areas [1]. As cities continue to expand, the presence of urban green spaces (e.g., parks, gardens, and street trees) becomes even more critical in ensuring urban sustainability and development [2]. Urban spaces provide numerous ecological, environmental, and cultural benefits to both the environment and the well-being of urban populations [3]. They not only provide a place for recreation and relaxation, but they also serve as habitats for a variety of plant and animal species and play an important role in controlling air quality, temperature, noise pollution, and general biodiversity in metropolitan areas [4,5]. Trees also enhance the value of properties in the cities, capture stormwater runoff, and reduce the energy required for cooling buildings during periods of high temperatures [6]. However, urban trees’ capacity to remove pollutants from the urban atmosphere largely depends on species traits (e.g., leaf area) [7]. Moreover, trees must possess physiological tolerance or phenotypic plasticity in their functional traits in order to survive and thrive in urban environments over the long term [8]. Hence, studying leaf functional traits (e.g., leaf size, shape, thickness, and chlorophyll content) of woody plants in urban green space environments is essential for understanding how these green spaces function and how they can be effectively managed and maintained sustainably. This kind of study can also help us determine tree species that are potentially more resilient to stresses that are commonly present in urban environments. 

Leaf functional traits of plants are the important bridge between plants and their environments, which can directly reflect the plant survival and adaptive strategies to the environment [9]. Plant morphological and physiological processes are extremely sensitive to environmental changes [10,11]. In particular, leaf shape and size correlate strongly with environmental conditions (e.g., temperature) from local to global scales [12,13]. The shape index, area, and thickness of leaves directly reflect a plant’s trade-off strategies in response to environmental stresses [14]. Plant functional traits (morphological, physiological, and phenological features), which are important indicators of plant growth and ecosystem dynamics, serve as a link between plants and environments. These traits determine a plant’s ability to acquire, use, and preserve resources in a particular environment [15]. For instance, Specific Leaf Area (SLA) is evident in the survival strategies that plants have developed to effectively deal with environmental fluctuations and optimize carbon assimilation [16].

Plant growth conditions of urban trees in most cities are deteriorating due to increasing urbanization rates and environmental stressors, such as water stress, high temperature, and increased ozone concentration [17]. Studies have demonstrated significant declines in the maximum photochemical quantum efficiency (Fv/Fm) and chlorophyll content of the different tree species in urban green spaces when ozone concentration and water shortage increase [18,19]. Dry air, high temperature, and soil conditions in cities or areas with comparable environmental conditions strongly affected stomatal conductance, water-use efficiency (WUE), and leaf water potential (Ψ_pd_, Ψ_md_) of woody trees [20,21]. Some tree species have strategies to cope with heat and drought stresses by decreasing Ψ_md_ and WUE [22]. However, considering Mongolia’s high temperature variations among seasons, information about the response of leaf functional traits of woody plants in Ulaanbaatar remains sparse to date.

Mongolia’s capital city, Ulaanbaatar, covers a total land area of 470,445.06 hectares. In 2017, the land use in the city was categorized into six different types, with agricultural land accounting for 50.20%, built-up areas for 14.78%, and forested areas for 15.75% of the urban land [23]. Ulaanbaatar is growing rapidly, with the human population reaching over 1.5 million in 2021, according to the National Statistics Committee [24]. This population growth coincides with an increase in urban green spaces in the capital city, which started in 2013. Many plants have been planted in various green spaces in the built-up areas of Ulaanbaatar. The dominant trees and shrubs planted in urban green zones are Salicaceae Lindl (7.79%), Ulmaceae Mirb (48.14%), and Leguminosae Juss (24.74%) [25]. Specifically, *Populus laurifolia* and *Ulmus pumila* have been widely planted in urban areas of Ulaanbaatar for a long time because of their high adaptability to poor environments, such as drought and degraded soil [26]. *Populus* species are considered pioneer species because of their fast growth rates and ability to inhabit large ranges [27]. Contrarily, *U. pumila* has a slow growth rate but is known for its high drought resistance, which stems from its natural habitat in the Mongolian desert [28].

Consequently, examining how their leaf functional traits respond to the urban environment will provide us with science-based insights into their adaptive mechanisms for coping with the harsh physical conditions in Ulaanbaatar.

In this study, we investigated the effects of different growing conditions on the morpho-physiological traits of *P. laurifolia* and *U. pumila* across five contrasting urban sites. It is hypothesized that there are significant variations in the morpho-physiological traits of *P. laurifolia* and *U. pumila* across five urban sites because of contrasting plant traits and study sites’ soil physicochemical characteristics. 

## 2. Results

### 2.1. Environmental Conditions

Soil chemical and physical properties were different among study sites, particularly at 20 cm soil depth. The dominant type of soil was sandy loam at all sites, indicating comparable values at all sites. The average pH ranges from 7.55–8.02, indicating slightly alkaline soil conditions across sites. Elements such as calcium, magnesium, potassium, and organic matter, which are the major elements of soil nutrients, had high values in the BE site. Soil calcium carbonate contents were similar across different sites (Table 1; Table S1).

### 2.2. Leaf Morpho-Physiological Traits

The interaction between study sites and leaf morphological traits was significant (*p* < 0.0001) in both species (Table S2). We found significant site effects on most of the morpho-physiological traits measured, except for the Ψ_md_ (Table S2).

**Figure 1:**
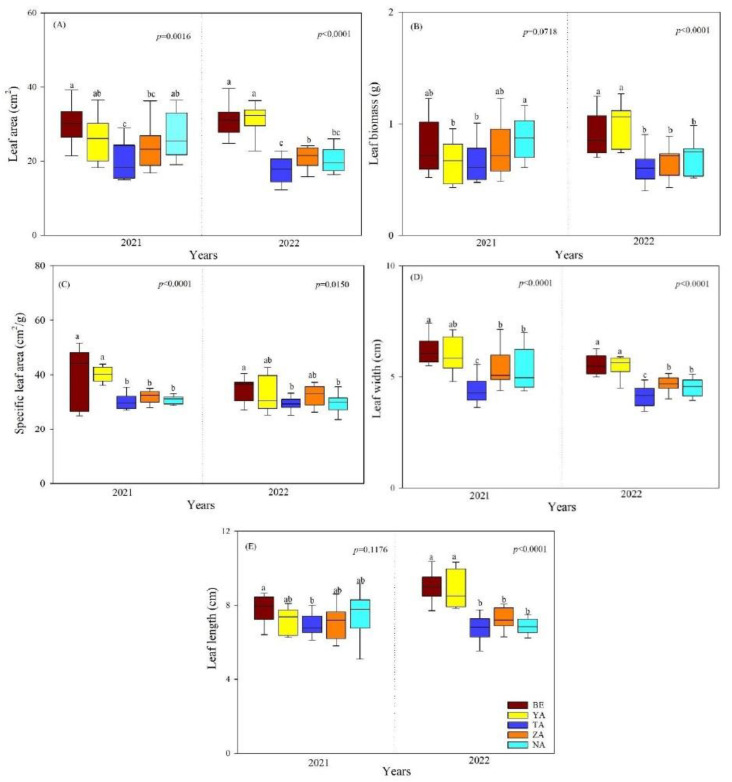
Leaf morphological traits of *P. laurifolia* across different urban study sites (BE-Beejin, YA-Yavuu, TA-Tasgan, ZA-Zanabazar, NA-Naadamchid) in Ulaanbaatar, Mongolia. Different lowercase letters indicate significant differences between study sites within the same year. (**A**) leaf area (LA); (**B**) leaf biomass (LB); (**C**) specific leaf area (SLA); (**D**) leaf width (LW); and (**E**) leaf length (LL).

The SLA, LL (*p* < 0.01), and LW (*p* < 0.0001) were significantly different between the study years, and a reverse pattern was observed for LA and LB of *P. laurifolia*. The mean LA, LB, SLA, LW, and LL of *P. laurifolia* were significantly lower in the TA site than in other sites. In contrast, in BE site, they were higher (Figure 1A–E). The LB, LA, SLA, LW, and LL of *P. laurifolia* were also highly significant between the study sites. Specifically, the SLA and LA were lower in 2022 than in 2021 (Figure 1B,C).

**Figure 2:**
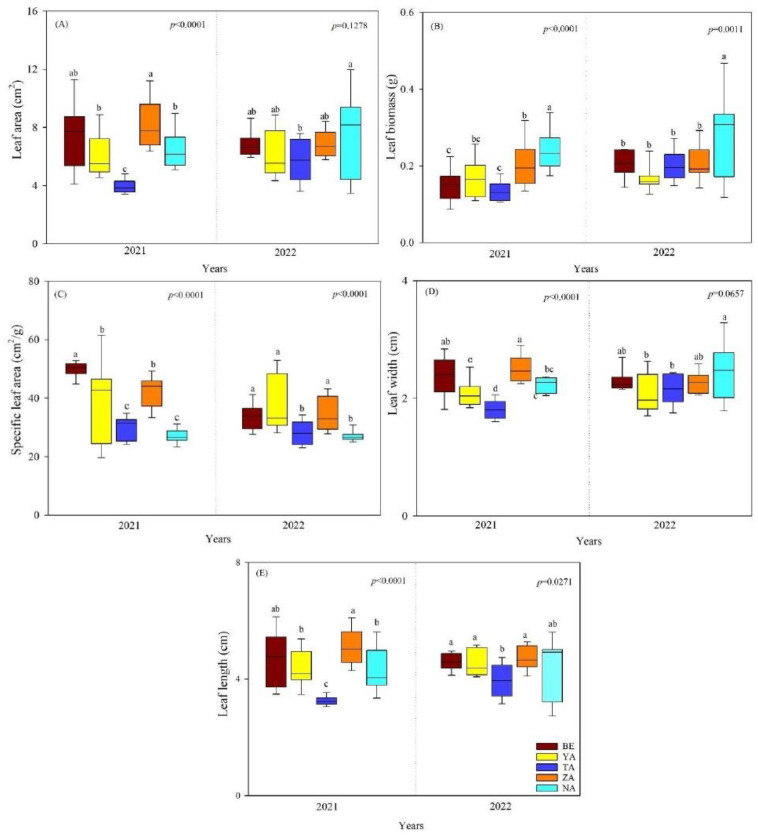
The leaf morphological traits of *U. pumila* across different urban study sites (BE-Beejin, YA-Yavuu, TA-Tasgan, ZA-Zanabazar, NA-Naadamchid) in Ulaanbaatar, Mongolia. Different lowercase letters indicate significant differences between study areas within the same year. (**A**) Leaf area (LA); (**B**) leaf biomass (LB); (**C**) specific leaf area (SLA); (**D**) leaf width (LW); and (**E**) leaf length (LL).

For *U. pumila*, LA, LW, and LL did not differ significantly between years, but LB and SLA were significantly different (*p* < 0.05). Additionally, LA, LB, LW, and LL were strongly and significantly different between sites in 2021 but showed significant decreases in 2022. Both in 2021 and 2022, the SLA was significantly different between sites (*p* < 0.0001, Figure 2). The leaf morphological traits of *U. pumila* were significantly lower in the TA site than in other sites (Figure 2).

**Figure 3:**
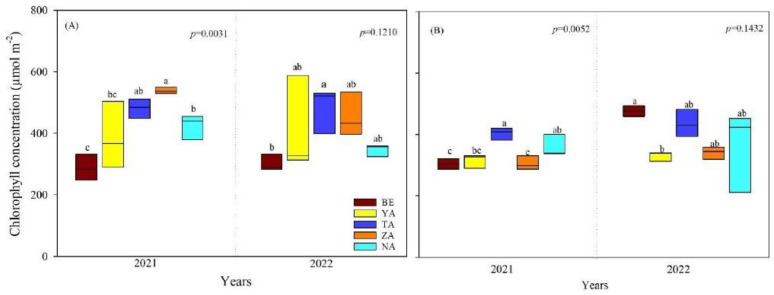
Leaf chlorophyll concentration of *P. laurifolia* (**A**) and *U. pumila* (**B**) growing in different urban study sites in Ulaanbaatar, Mongolia. Different lowercase letters indicate significant differences among sites.

The leaf chlorophyll concentration of *P. laurifolia* and *U. pumila* ranged from 294.58 to 496.87 µmol m^−2^ and significantly varied between sites (*p* < 0.05, Table S1, Figure 3). The leaf chlorophyll concentration of both species differed significantly between sites only in 2021 (*p* < 0.05). Specifically, the leaf chlorophyll concentration of *P. laurifolia* at the ZA site was the highest, whereas *U. pumila* had a lower concentration. Contrarily, the leaf chlorophyll concentration of *P. laurifolia* growing in the BE site was the lowest.

Table 2 shows an overview of the measured mean values of Fv/Fm and PI_abs_ parameters. Overall, both Fv/Fm and PI_abs_ tended to be slightly lower in 2022 compared to 2021 for both species, regardless of site.

**Figure 4:**
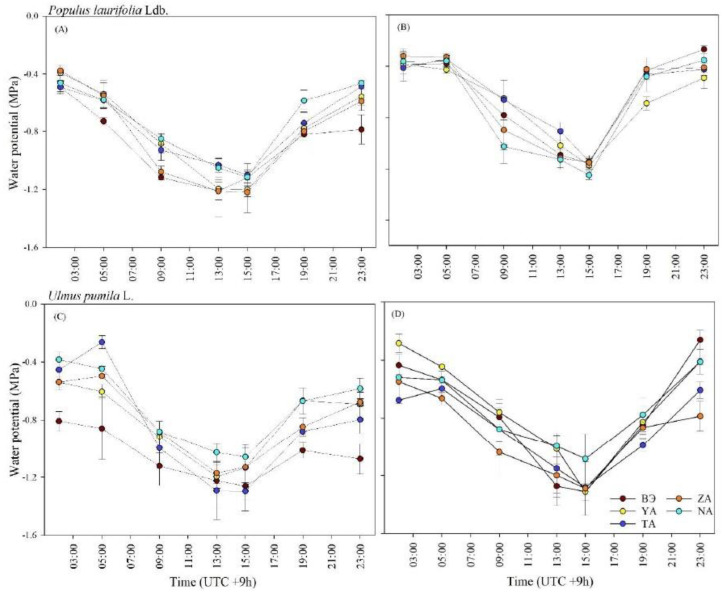
Diurnal variation in leaf water potential of *P. laurifolia* (**A**,**B**) and *U. pumila* (**C**,**D**) growing in the urban study sites measured in July 2019 and 2020 in Ulaanbaatar, Mongolia. Vertical bars represent standard error.

In both species, the lowest declines in water potential detected in all sites were at 13:00 and 15:00 in 2019 and 2020, respectively (Figure 4). The trees in the study sites experienced water deficits at 09:00 and gradually recovered from these deficits after 17:00.

In all species, the leaf predawn water potential (Ψ_pd_) was significantly different between years (*p* < 0.01) and sites (*p* < 0.05, Table 3). The Ψ_pd_ of *P. laurifolia* leaves showed significant decreases in all sites in 2021 compared to 2022, but even in these cases, the value of Ψ_pd_ did not fall below −0.71 MPa. In TA, Ψ_pd_ and Ψ_md_ decreased significantly in *U. pumila* in 2021 compared to 2022.

### 2.3. Phenotypic Plasticity Index of Morphological and Physiological Traits 

In this study, we demonstrated that the morphological traits and the mean phenotypic plasticity index (PPI) of leaf biomass for all species were higher compared to other traits. Additionally, the physiological traits and performance index (PI_abs_) exhibited greater plasticity in both species (Table 4). They displayed different phenotypic plasticity responses across the study sites. In *P. laurifolia*, the mean PPI for morphological traits (0.21) and physiological traits (0.17) were similar. However, in *U. pumila*, the mean PPI for morphological traits (0.20) was lower than that for physiological traits (0.21).

## 3. Discussion

### 3.1. Variation in Leaf Morphological Traits across Different Urban Green Spaces 

Environmental conditions directly and indirectly influence plant function by affecting various traits [29]. Trees were sensitive to the urban environment and adapted to the environmental changes by adjusting their leaf functional traits. Some studies have shown that leaf length, leaf width, and leaf area all increase with more shade, making their structure and function adapt to the changing environment [30,31]. In this study, the mean values of LA, LB, and SLA of *P. laurifolia* in study sites were BE > YA > NA > ZA > TA, and this result agrees with our hypothesis (i.e., there are significant variations in morphological traits of *P. laurifolia* across sites). The result can be explained by variations in environmental conditions. *P. laurifolia* trees growing in BE and YA have higher tree heights and thus have greater light access compared to those in the other sites, resulting in a competitive advantage for the species. In general, taller trees receive more sunlight exposure, which allows them to develop larger leaves and a wider canopy [32]. This can be supported by increased SLA since larger leaves have thinner leaves, making them more efficient at capturing light and conducting photosynthesis in changing light conditions [33]. Moreover, a higher LA, LB, and SLA of *P. laurifolia* can be due to a higher organic matter content in BE than in other sites. A higher organic matter content can result in increased LA and SLA due to the availability of essential macro and micronutrients, improved soil structure and moisture retention, and the promotion of root growth and development [34,35]. The P_2_O_5_ level, which is critical to leaf metabolic processes and growth [36], was also significantly higher in BE and YA sites. The availability of phosphorus in a particular area influences leaf expansion biomass accumulation and root development [37]. This can explain the observed significant effect of year (time) on the specific leaf area of *P. laurifolia.* Changes in SLA over years suggest that *P. laurifolia* adjusts its leaf growth in response to phosphorus levels.

In the case of *U. pumila*, most of the measured morphological traits were greater in ZA and lower in TA. Similarly, the *U. pumila* trees in ZA were 5% taller than those in the TA site, which explains their higher morphological traits. The ZA site also has 30% higher OM compared to TA. The richness of OM in ZA may have resulted in a high macronutrient supply (e.g., nitrogen) due to improved soil nutrient retention, which is important for leaf and overall plant growth development [38]. Higher OM in ZA may have also stimulated root growth due to enhanced soil structure, which may have supported better leaf expansion and growth.

Specific leaf area is an important plant functional trait, as it is an indicator of ecophysiological characteristics, such as relative growth rate, stress tolerance, and leaf longevity [39]. In urban environments, the urban heat island effect, drought, and heavy metal exposure tend to decrease SLA, while high-nutrient soils, increased light levels, and disturbances tend to increase SLA [40]. This further explains the observed lower SLA of both *P. laurifolia* and *U. pumila* in more exposed study sites (e.g., TA and NA). Soil in more exposed areas or with less tree cover may have low nutrient and organic matter content, causing trees to experience greater stress [15]. Trees in these areas may allocate less carbon to leaf production, thereby reducing leaf area and biomass [9]. SLA and LA have a significant relationship with soil fertility, as soil plays a fundamental role in the performance of plant functional traits [41].

### 3.2. Variation in Leaf Physiological Traits and Phenotypic Plasticity Index across Contrasting Sites 

Here, *U. pumila* also had a higher PPI for physiological traits than morphological traits, suggesting that the species exhibited more significant plasticity in its physiological responses than in its morphological characteristics across sites. The result further implies that while *U. pumila* can adjust to environmental stresses in the urban area, its leaf morphological traits are less responsive or less flexible. The higher PPI in *U. pumila* could allow the species to grow and survive in harsh conditions typical of urban environments. Specifically, our results demonstrate that *U. pumila* in the NA site had a higher average performance index (PI_abs_) than other sites, possibly indicating lower overall stress and higher photosynthetic capacity [42]. The results suggest that the prevailing environmental conditions at the NA site are favorable for the species. This can lead to more efficient physiological performance and efficiency in photosynthesis. Moreover, the chlorophyll fluorescence parameters and leaf traits of some species varied by site type [43]. In our study, we found that the chlorophyll concentration of *U. pumila* varied significantly between different sites. This variation also indicates that the species is highly responsive to changing environmental conditions, exemplifying its physiological plasticity.

Leaf water potential as a direct index can be used to indicate the water conditions and the drought stress degree of plants in urban environments [44]. In the present study, the declines in Ψ of the two tree species in all sites, particularly during the 13:00 to 15:00 time periods, suggest that both species were highly vulnerable to water stress during the warmest part of the day in the study sites [34,35]. Our results also found that Ψ of both species can be reversed to normal conditions, under which the species are expected to exhibit their regular physiological functions. This means that the species can effectively recover Ψ from diurnal water stress (e.g., increased transpiration rates, soil moisture deficits). This result was consistent with the result of our previous study [45]. Such a recovery can be made through various mechanisms, including stomatal regulation and increased root water uptake.

## 4. Materials and Methods

### 4.1. Tree Species and Study Sites 

**Figure 5:**
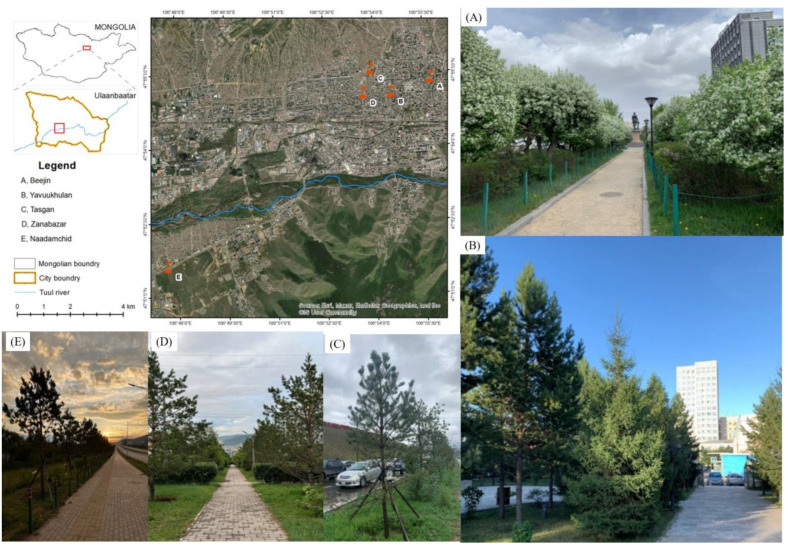
Location of the five contrasting urban sites (background image from Google Earth) showing (**A**) Beejin, (**B**) Yavuu, (**C**) Tasgan, (**D**) Zanabazar, and (**E**) Naadamchid sites in Ulaanbaatar, Mongolia.

The study was in Ulaanbaatar (47°94′23.58″ N, 106°90′15.04″ E), which is the capital and the political, economic, and cultural center of Mongolia (Figure 5). It has a moist and cold summer and a harsh winter climate, with a mean annual temperature of −3.1–1.5 °C. The mean elevation of the city area is 1350 masl. 

**Figure 6:**
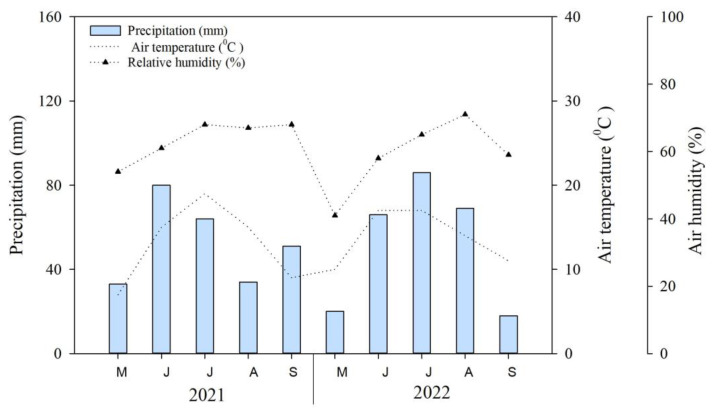
Mean monthly air temperature (broken line), precipitation (bar), and air humidity (broken dotted line) in the study area in Ulaanbaatar, Mongolia (i.e., May–September 2021–2022).

The mean annual precipitation is about 250 mm, with over 80% occurring from June to September. During growing seasons, the average air temperature and precipitation of growing seasons (May–September) in 2021–2022 were 13.0–13.7 °C and 51.8–52.6 mm, respectively (Figure 6). The precipitation in July of 2022 exceeded that of July 2021 (c.a., 10–15%). The 2021–2022 meteorological data were obtained from the nearest weather station [46], which is approximately five kilometers away from the study site. The mean non-frost period is 90–110 days [47]. Moreover, the study area is characterized by dark kastanozem soil [48,49] and mollic leptosols by international soil classification [50].

Three subplots were established at each site, with an area of 10 m × 30 m and a distance of 5 m between plots. In this study, we selected two woody plants (*P. laurifolia* and *U. pumila*) in five sites (Table 5). In urban green areas in Ulaanbaatar, they are among the most common tree species due to their fast growth rate [51,52].

### 4.2. Soil Analysis 

Soil analyses [53] were performed for each site before sampling and measuring plant morpho-physiological traits. Soil samples were taken to a depth of 0–20 cm (0–10 cm, 10–20 cm) at each urban site for the analysis of physicochemical properties (Table 1). The soil samples were air-dried, sieved through a 2 mm sieve, and stored at room temperature. Soil pH was determined on a 1:2.5 air-dried soil/distilled water mixture using a glass electrode pH meter [54]. Calcium carbonate content was determined by the volumetric method [55]. Available phosphorus (P_2_O_5_) was measured by molybdenum blue colorimetry after (NH_4_)_2_CO_3_ digestion [56]. Nitrate-nitrogen (NO_2_-N) was determined using a CH_3_COONa digestion and Spectro colorimetry. Potassium (K_2_O) was analyzed by flame spectrometry [57].

### 4.3. Leaf Morphology and Physiological Trait Analysis 

The research was conducted over two growing seasons (2021 and 2022). For the measurement of leaf morpho-physiological traits, three mature and healthy individuals from each species were randomly selected from each site. All measurements were taken on clear days of the growing season. From three individuals of each species, twenty healthy and fully expanded leaves were chosen for measuring the leaf area (LA), leaf length (LL), leaf width (LW), leaf biomass (LB), and specific leaf area (SLA). Immediately after collection, the leaves were sealed in a plastic zip-lock bag and stored in a cold container to avoid water loss during transportation until further processing in the laboratory. The leaves were photo-scanned using an HP LaserJet scanner (M1132 MFP, ID, USA) with a 600 dpi resolution, and ImageJ v1.52 (National Institutes of Health, Bethesda, MD, USA) processing software was used to measure leaf morphological parameters following the procedure in Schneider et al. [58] and the modified procedure by Hernandez et al. [59].

The fresh weight of the leaves was weighed using a high-precision electronic scale (d = 0.001 g, Discovery Semi-Micro and Analytical Balance, Ohaus Corp., Nanikon, Switzerland), and they were dried at 80 °C for 48 h until they were a constant dry weight. The SLA was determined using the ratio of leaf area to leaf dry weight (SLA = LA (cm^2^)/LDM (g); [60]).

The Ψ_pd_ was measured before sunrise and Ψ_md_ at solar noon following the procedures in Scholander et al. [61], and the Ψ_pd_ and Ψ_md_ (MPa) were measured on healthy, fully expanded, and sun-exposed apical leaves using a pressure chamber (Model 1505D EXP, PMS Instrument Company, Albany, OR, USA) (c.a. 1.5 m above the ground). The leaf chlorophyll concentration was measured by an MC-100 chlorophyll concentration meter (Apogee Instruments, Inc., Logan, UT, USA) at midday [62].

In the assessment of chlorophyll fluorescence indicators, we focused on the values of Fv/Fm and PI_abs_ physiological parameters measured on two species grown at five sites. Chlorophyll fluorescence measurements were performed using the Handy-PEA continuous excitation plant efficiency analyzer (Hansatech Instruments Ltd., King Lynn, UK) in 2021 and 2022. The leaf clips were applied to the leaves 20 min (i.e., based on our pilot experiments) before the measurements were taken to allow for dark adaptation. The light pulse intensity used was 3000 µmol m^−2^ s^−1^ for 1 s. The maximum quantum yield of PS II photochemistry Fv/Fm and performance index were calculated according to Guidi et al. [63]. Moreover, the leaf performance index (PI_abs_) of each species was determined in the present study. The performance index (PI_abs_) is a parameter thought to be sensitive to various types of stress. It is widely used to compare primary photochemical reactions [64].

### 4.4. Phenotypic Plasticity Index and Data and Statistical Analyses 

Here, we made sure that all instruments used to measure all the parameters were calibrated against standardized references. Potential outliers were identified using boxplots and subsequently verified by cross-referencing the results with the original raw data. For all studied traits, a phenotypic plasticity index (PPI) was calculated using the following formula: PPI = (maximum mean value − minimum mean value)/(maximum mean value). The index varies from zero to one and allows comparisons among traits with different units [65]. The phenotypic plasticity index (PPI) is one of the important indicators that reflects the conservation and heritability of traits in the evolutionary process. The higher the value of the indicator, the stronger the adaptability of plants to the environment [31]. 

Before statistical analysis, we conducted the Shapiro–Wilk test to test whether our data follow a normal distribution. One-way analysis of variance (ANOVA) was employed to determine the effects of sites on the morpho-physiological traits measured for each species. To assess multiple comparisons among the treatments, Duncan’s multiple range test (DMRT) was used. All the statistical analyses were conducted by the Statistical Analysis Software (SAS, version 9.4) package [66].

## 5. Conclusions

The present study generally demonstrated that growing environment conditions in urban green spaces significantly influenced the leaf morpho-physiological functional traits of both *P. laurifolia* and *U. pumila*. In conclusion, we revealed significant variations in the morpho-physiological traits of *P. laurifolia* and *U. pumila* across the five urban sites. These variations reflect the effects of local environmental conditions in urban spaces in Mongolia. However, the limitations of the present study, such as a limited number of sites and only two tree species included, may have influenced the overall results and conclusions drawn. Other plant traits (e.g., wood traits), which are good indicators of a plant’s responses to urban environments, could also have a substantial influence on the results. Hence, long-term, large-scale studies are recommended to examine how diverse species respond to different urban growing conditions and to include other ecologically important plant traits for a better understanding of urban trees in a changing environment.

## Figures and Tables

**Table 1:** Soil physicochemical properties of the contrasting study sites in Ulaanbaatar, Mongolia. Values represent the mean of soil depth (*n* = 3 ± SE). Different lowercase letters indicate significant differences across the treatments at α = 0.05.

Site	BE	YA	TA	ZA	NA
pH	7.55 ± 0.05 ^c^	7.75 ± 0.04 ^bc^	7.79 ± 0.08 ^b^	7.67 ± 0.09 ^bc^	8.02 ± 0.03 ^a^
Organic matter	4.00 ± 0.23 ^a^	2.66 ± 0.16 ^b^	1.88 ± 0.43 ^c^	2.78 ± 0.08 ^b^	1.43 ± 0.01 ^c^
CaCO_3_	0.91 ± 0.45 ^a^	0.73 ± 0.36 ^a^	1.05 ± 0.53 ^a^	0.95 ± 0.03 ^a^	0.95 ± 0.13 ^a^
N-NO_3_	7.52 ± 1.10 ^a^	3.72 ± 0.32 ^b^	5.15 ± 0.57 ^ab^	6.80 ± 1.43 ^a^	5.78 ± 0.39 ^ab^
P_2_O_5_	4.73 ± 0.54 ^a^	3.73 ± 0.18 ^a^	1.41 ± 0.08 ^b^	1.44 ± 0.04 ^b^	0.78 ± 0.09 ^b^
K_2_O	16.15 ± 1.18 ^ab^	12.00 ± 0.17 ^b^	12.45 ± 0.95 ^b^	14.30 ± 1.39 ^ab^	17.00 ± 1.96 ^a^
Ca	0.58 ± 0.01 ^a^	0.55 ± 0.06 ^a^	0.49 ± 0.01 ^a^	0.56 ± 0.02 ^a^	0.48 ± 0.01 ^a^
Mg	0.32 ± 0.03 ^a^	0.28 ± 0.02 ^ab^	0.21 ± 0.04 ^b^	0.28 ± 0.02 ^ab^	0.24 ± 0.02 ^b^
Na	0.29 ± 0.02 ^a^	0.16 ± 0.01 ^b^	0.26 ± 0.02 ^ab^	0.29 ± 0.08 ^a^	0.16 ± 0.02 ^b^
K	0.06 ± 0.00 ^a^	0.04 ± 0.00 ^b^	0.03 ± 0.00 ^b^	0.04 ± 0.00 ^b^	0.03 ± 0.00 ^b^

**Table 2:** Maximum quantum yield of photochemistry (Fv/Fm) and performance index (PI_abs_) measured from *P. laurifolia* and *U. pumila* across different study sites in Ulaanbaatar, Mongolia. Different lowercase letters indicate significant differences among study sites at α = 0.05.

Species	Sites	Fv/Fm		PI_abs_	
		July 2021	July 2022	July 2021	July 2022
*P. laurifolia*	BE	0.85 ± 0.00 ^a^	0.81 ± 0.01 ^b^	3.95 ± 0.44 ^a^	1.52 ± 0.13 ^c^
	YA	0.85 ± 0.01 ^a^	0.84 ± 0.00 ^a^	6.01 ± 1.62 ^a^	3.22 ± 0.24 ^ab^
	TA	0.82 ± 0.02 ^a^	0.82 ± 0.00 ^b^	8.17 ± 4.83 ^a^	3.96 ± 0.32 ^a^
	ZA	0.83 ± 0.01 ^a^	0.77 ± 0.01 ^c^	5.01 ± 0.46 ^a^	2.69 ± 0.32 ^b^
	NA	0.84 ± 0.00 ^a^	0.83 ± 0.00 ^ab^	5.02 ± 0.57 ^a^	3.49 ± 0.36 ^ab^
*U. pumila*	BE	0.77 ± 0.03 ^a^	0.81 ± 0.01 ^ab^	2.42 ± 1.09 ^ab^	2.55 ± 0.52 ^b^
	YA	0.72 ± 0.02 ^a^	0.77 ± 0.02 ^c^	1.03 ± 0.24 ^b^	2.11 ± 0.38 ^b^
	TA	0.69 ± 0.12 ^a^	0.83 ± 0.00 ^a^	2.89 ± 0.47 ^a^	2.60 ± 0.22 ^b^
	ZA	0.74 ± 0.02 ^a^	0.79 ± 0.00 ^bc^	1.80 ± 0.41 ^ab^	3.06 ± 0.24 ^b^
	NA	0.84 ± 0.01 ^a^	0.83 ± 0.01 ^a^	3.23 ± 0.27 ^a^	4.17 ± 0.22 ^a^

**Table 3:** Mean predawn and midday leaf water potentials of *P. laurifolia* and *U. pumila* across contrasting study sites in Ulaanbaatar, Mongolia. Different letters indicate the significant differences in the mean values among study sites and research years for species at α = 0.05.

Species		Sites	July 2021	July 2022
*P. laurifolia*	Ψ_pd_	BE	−0.71 ^b^	−0.26 ^b^
		YA	−0.51 ^a^	−0.28 ^b^
		TA	−0.52 ^a^	−0.14 ^a^
		ZA	−0.44 ^a^	−0.23 ^ab^
		NA	−0.39 ^a^	−0.19 ^ab^
	Ψ_md_	BE	−1.16 ^b^	−0.93 ^a^
		YA	−0.90 ^a^	−0.97 ^a^
		TA	−1.00 ^ab^	−1.01 ^a^
		ZA	−0.94 ^ab^	−0.95 ^a^
		NA	−0.99 ^ab^	−0.90 ^a^
*U. pumila*	Ψ_pd_	BE	−0.41 ^a^	−0.50 ^ab^
		YA	−0.65 ^bc^	−0.58 ^b^
		TA	−0.78 ^c^	−0.41 ^a^
		ZA	−0.58 ^ab^	−0.40 ^a^
		NA	−0.43 ^a^	−0.53 ^b^
	Ψ_md_	BE	−1.09 ^a^	−1.10 ^a^
		YA	−1.07 ^a^	−1.17 ^a^
		TA	−1.16 ^a^	−1.04 ^a^
		ZA	−1.08 ^a^	−0.91 ^a^
		NA	−0.89 ^a^	−0.95 ^a^

**Table 4:** Index of phenotypic plasticity (mean value of study years) of leaf morphological and physiological traits of *P. laurifolia* and *U. pumila* across contrasting study sites in Ulaanbaatar, Mongolia. Different letters indicate the significant differences in the mean values among study sites at α = 0.05.

Species	Site	Morphological Traits					Physiological Traits			
		LA	LB	SLA	LL	LW	Chl	Fv/Fm	PI_abs_	WP_(md)_
*P. laurifolia*	BE	0.30 ^a^	0.33 ^a^	0.12 ^a^	0.21 ^a^	0.14 ^a^	0.14 ^b^	0.02 ^a^	0.25 ^b^	0.12 ^b^
	YA	0.25 ^a^	0.25 ^a^	0.10 ^a^	0.10 ^b^	0.20 ^a^	0.32 ^a^	0.04 ^a^	0.37 ^ab^	0.07 ^b^
	TA	0.30 ^a^	0.33 ^a^	0.12 ^a^	0.17 ^ab^	0.18 ^a^	0.10 ^b^	0.06 ^a^	0.59 ^a^	0.12 ^b^
	ZA	0.29 ^a^	0.29 ^a^	0.11 ^a^	0.15 ^ab^	0.19 ^a^	0.09 ^b^	0.04 ^a^	0.35 ^b^	0.35 ^a^
	NA	0.28 ^a^	0.32 ^a^	0.16 ^a^	0.16 ^ab^	0.21 ^a^	0.08 ^b^	0.02 ^a^	0.27 ^b^	0.08 ^b^
*U. pumila*	BE	0.31 ^a^	0.35 ^a^	0.12 ^ab^	0.15 ^a^	0.18 ^a^	0.04 ^b^	0.10 ^a^	0.76 ^a^	0.13 ^a^
	YA	0.29 ^a^	0.31 ^a^	0.19 ^a^	0.15 ^a^	0.18 ^a^	0.07 ^b^	0.11 ^a^	0.61 ^ab^	0.23 ^a^
	TA	0.24 ^a^	0.25 ^a^	0.09 ^b^	0.14 ^a^	0.13 ^a^	0.07 ^b^	0.02 ^a^	0.45 ^bc^	0.11 ^a^
	ZA	0.23 ^a^	0.28 ^a^	0.12 ^ab^	0.16 ^a^	0.14 ^a^	0.08 ^b^	0.08 ^a^	0.55 ^ab^	0.14 ^a^
	NA	0.23 ^a^	0.29 ^a^	0.12 ^ab^	0.18 ^a^	0.13 ^a^	0.21 ^a^	0.03 ^a^	0.27 ^c^	0.08 ^a^

**Table 5:** Characteristics of each urban site and tree species in Ulaanbaatar, Mongolia.

Urban Sites	Description	Tree Species			
		*P. laurifolia*		*U. pumila*	
		H (m)	D (cm)	H (m)	D (cm)
Beejin (BE)	This site is located in the middle of main roads (47°55′22.78″ N, 106°55′43.74″ E) and with high-rise buildings around, which serve to buffer people from traffic. The area is 7000 m^2^. The trees and shrubs were planted in rows with a distance of 3–5 m between them. The study area was dominated by *P. laurifolia*, *U. pumila*, and *Pinus sylvestris*. The other dominant trees species were *Picea obovata* and *Acer negundo* L. The soil has a sandy mechanical composition, a pH of 7.55, and comprises 4.00% organic matter.	5.05	43.17	0.8	3.97
Yavuu (YA)	The site is a small park that is located among high-rise buildings (47°55′5.61″ N, 106°54′32.01″ E), and the area is 4500 m^2^; the trees and shrubs are in rows and groups with a distance of 3–5 m between them. The study area was also dominated by *P. laurifolia*, *U. pumila*, and *P. sylvestris*. The next dominant tree species were *Picea obovata* and *Larix sibirica.* The soil is generally sandy, has a pH of 7.75, and comprises 2.66% organic matter.	4.97	43.00	0.87	4
Tasgan (TA)	The site is in the middle of the main road (47°55′34.03″ N, 106°53′55.85″ E), with a building on the front side and a residential area (per district) on the north side. The area is about 5000 m^2^. The trees and shrubs were planted in rows with a distance of 3–5 m between them. The study area was dominated by *P. laurifolia*, *U. pumila*, and *P. sylvestris.* The predominant tree species was *Syringa josikaea.* The soil is sandy, with a pH of 7.79, and comprises 1.88% organic matter.	3.88	23.54	1.35	3.51
Zanabazar (ZA)	The site is in the middle of a residential area (ger district, 47°55′4.74″ N, 106°53′41.28″ E), where the trees were planted in rows with 3–5 m distance in between, and shrubs were planted in groups. The study area was dominated by *P. laurifolia*, *U. pumila*, and *P. sylvestris.* The predominant tree species were *L. sibirica* and *Syringa josikaea.* The area is 6000 m^2^. The soil is sandy, with a pH of 7.67, and comprises 2.78% organic matter.	3.02	20.87	1.52	5.38
Naadamchid (NA)	The site is located along the main road (47°51′38.05″ N, 106°47′40.49″ E). The trees were planted in rows with a distance of 3–5 m between them. This area is about 2800 m^2^. The study area was dominated by *P. laurifolia*, *U. pumila*, and *P. sylvestris*. The predominant tree species is *Malus baccata L*. The soil texture is sandy, with a pH of 8.02, and comprises 1.43% organic matter.	3.05	25.49	2.78	5.82

H—mean height; D—mean breast diameter.

## Data Availability

The data used are primarily reflected in the article. Other relevant data are available from the authors upon request.

## Supplementary Materials

The following supporting information can be downloaded at: https://www.mdpi.com/article/10.3390/plants13192709/s1, Table S1: Different soil physical and chemical properties of study sites; Table S2: Impact of site conditions on leaf functional traits of tree species.
