# Supplementary material for: Variation in Leaf Functional Traits of Populus laurifolia Ldb and Ulmus pumila L. Across Five Contrasting Urban Sites in Ulaanbaatar, Mongolia
Source: Plants (Basel). 2024 Sep 27;13(19):2709. doi: 10.3390/plants13192709 (PMC11478421; doi:10.3390/plants13192709)
Supplement: Supplementary file 1 [file plants-13-02709-s001.zip › plants-3179674-supplementary.pdf]

Supplementary Materials

Table S1

Different of soil physical and chemical properties of study sites

Measurements	D	F	P value
pH	4	7.79	0.0041*
Organic matter	4	18.29	<.0001***
$CaCO_3$	4	0.11	0.9747
$N\text{-}NO_3$	4	2.84	0.082
P_2O_5	4	19.33	<.0001***
K_2O	4	2.99	0.073
Ca	4	2.29	0.1312
Mg	4	3.07	0.0687
Na	4	3.52	0.0484*
K	4	13.5	0.0005**

Notes: Significant effects are indicated as follows: ***p < 0.0001**p < 0.01 *p < 0.05

Table S2

Impact of sites condition on leaf functional traits of tree species

Measurements	*P. laurifolia*			*U. pumila*	
	D	F	P value	F	P value
Leaf area, cm^2	4	22.33	<.0001***	10.4	<.0001***
Leaf biomass, gr	4	4.85	0.0012**	11.83	<.0001***
Specific leaf area, cm^2/gr	4	12.91	<.0001***	20.74	<.0001***
Leaf width, cm	4	25.02	<.0001***	10.27	<.0001***
Leaf length, cm	4	15.56	<.0001***	13.77	<.0001***
Leaf water potential (Ψ_{pd}), MPa	4	3.25	0.1140	4.51	0.0106*
Leaf water potential (Ψ_{md}), MPa	4	1.47	0.3373	1.83	0.1664
Chlorophyll concentration, µmol m^2	4	7.73	0.0006**	4.59	0.0107*
Chlorophyll fluorescence, µmol $m^{-2} s^{-1}$	4	6.24	0.0002**	1.32	0.2718
Performance index,	4	1.08	0.3700	8.47	<.0001***

Notes: Significant effects are indicated as follows: ***p < 0.0001**p < 0.01 *p < 0.05
